# Comparing Traditional and Toxin-Oriented Approaches towards Antivenom Production against *Bitis arietans* Snake Venom

**DOI:** 10.3390/toxins15090584

**Published:** 2023-09-20

**Authors:** Felipe Raimondi Guidolin, Kemily Stephanie de Godoi, Angela Alice Amadeu Megale, Cristiane Castilho Fernandes da Silva, Roberto Tadashi Kodama, Daniela Cajado-Carvalho, Leo Kei Iwai, Patrick Jack Spencer, Fernanda Calheta Vieira Portaro, Wilmar Dias da Silva

**Affiliations:** 1 Immunochemistry Laboratory, Butantan Institute, São Paulo 05503-900, Brazil; kemilysgodoi@gmail.com (K.S.d.G.); angela.aamadeu@gmail.com (A.A.A.M.); 2 Laboratory of Structure and Function of Biomolecules, Butantan Institute, São Paulo 05503-900, Brazil; crisscastilho@gmail.com (C.C.F.d.S.); pararoberval@gmail.com (R.T.K.); fernanda.portaro@butantan.gov.br (F.C.V.P.); 3 Laboratory of Applied Toxinology (LETA), Center of Toxins, Immune-Response and Cell Signaling (CeTICS), Butantan Institute, São Paulo 05503-900, Brazil; danicajado@gmail.com (D.C.-C.); leo.iwai@butantan.gov.br (L.K.I.); 4 Nuclear and Energy Research Institute, University of São Paulo, São Paulo 05508-000, Brazil; patrickfrombrazil@gmail.com

**Keywords:** snake venom, antivenom, antibody, sub-Saharan Africa, *Bitis*, *Bitis arietans*

## Abstract

Accidents with snakes are responsible for about 32,000 deaths annually in sub-Saharan Africa, caused mostly by snakes from the genus *Bitis*, in particular *Bitis arietans*. *B. arietans* venom is composed of a complex mixture of toxins, mainly metalloproteases, serine proteases, phospholipases, lectins, and disintegrins. In this work, we compared two approaches to anti-*B*. *arietans* antivenom production: immunization with crude snake venom (“traditional approach”) and immunization with selected key toxins isolated from the snake venom (“toxin oriented” approach). Fractions from *B. arietans* venom were isolated by size exclusion chromatography. Crude venom and samples containing serine proteases or metalloproteases were selected for the immunization of BALB/c mice. Anti-*B. arietans* and anti-serine proteases plasmas showed a similar recognition profile and higher titers and affinity than the anti-metalloproteases plasma. Cross-recognition of other *Bitis* venoms was observed, but with low intensity. Although the plasma of all experimental groups inhibited the enzymatic activity of *B. arietans* venom in vitro, in vivo protection was not achieved. Our results have shown limitations in both approaches considered. Based on this, we proposed a model of polyclonal, species-specific, monovalent antivenoms that could be used as a base to produce customizable polyvalent sera for use in sub-Saharan Africa.

## 1. Introduction

Snakebites are a public health concern in many countries, especially in Latin America, South and Southeast Asia, and sub-Saharan Africa [1,2], and were included in the neglected tropical diseases list in 2017 [3]. In sub-Saharan Africa, it is estimated that 300,000 accidents happen each year, resulting in 32,000 deaths and over 9000 complications, such as amputations and local, permanent lesions [1]. Snakebite is related to field labor, as 95% of the accidents happen in rural areas and involve young men with ages below 15 [4], and bites usually occur in the hands, feet, and ankles [5]. Even when death is prevented, permanent disability can make it impossible to work, with great economic impacts at a personal and familiar level [1]. 

Antivenoms were provided to Africa by three companies: Aventis Pasteur (based in Lyon, France), Behringwerke (based in Marburg, Germany), and the South African Institute for Medical Research (SAIMR, based in Johannesburg, South Africa). However, by the end of the 1990s, the two European companies had ceased production due to financial reasons, with SAIMR’s production alone not being sufficient to cover continental demand [6]. The supply crisis forced local governments to buy antivenoms from India, which produces serum against its own local snakes and is therefore ineffective in neutralizing the venom of endemic African species [7]. The low quality of available antivenoms, their high cost, and negative treatment results have led to distrust by the population, and many chose to be treated instead by traditional faith healers, dying before proper medical treatment could be administered [1,5].

The African continent is home to a diverse herpetofauna, with over 400 snake species, of which about 30 are involved in human accidents [8]. Snakes from the *Bitis* genus are widespread and are responsible for most envenomation cases, with *Bitis arietans* alone being involved in more accidents and deaths than all other African snakes together [9,10]. *Bitis* envenoming is characterized by local effects such as local hemorrhage, necrosis, and compartmental syndrome and systemic effects such as thrombocytopenia, consumption coagulopathy, and persistent hypotension [9,11]. In general, *Bitis* venom is composed mainly of proteins from seven families: metalloproteases (SVMPs), serine proteases (SVSPs), disintegrins, C-type lectins, phospholipase A_2_, Kunitz inhibitors, and cystatins [12,13,14,15,16]. SVMPs and SVSPs are the main components, representing between 40% and 50% of dry venom weight [14,16]. The venom toxins affect the coagulation cascade in diverse ways. SVMPs are mostly anticoagulants and can directly attack the endothelium of blood vessels [17] or inhibit platelet aggregation [18]. SVMPs can be categorized into three classes [19] according to their complexity: PI (which contains the protease domain), PII (which contains the protease and disintegrin domains), and PIII (which contains the protease and disintegrin domains and cysteine-rich regions). SVSPs are among the most well-studied snake toxins [20]. They can be classified as trypsin-like enzymes [12], with a mostly procoagulant action [21]. Disintegrins and lectins are non-enzymatic polypeptides that affect platelet aggregation [22,23].

Antivenoms are, to date, the only specific treatment for snakebites and have been used since the end of the 19th century, in great part due to the production methodology proposed by Vital Brazil in 1889 [24]. In short, serum-producing animals (usually horses) are immunized with an antigenic mixture containing a pool of crude venom from different snake species within the same genus. About 15 to 20 days after inoculation, blood is collected, and the antibodies present in the plasma are purified and processed, becoming the anti-ophidic serum [25]. Although successful, this method is out-of-date considering the many advantages made in the fields of venomics and proteomics. Discoveries in these fields have revealed important information about venom composition and the role of each toxin in envenomation and have provided the basis for new serum production methodologies to emerge. Monoclonal antibodies have been used to isolate and characterize specific venom components [26], recognize and neutralize toxins [27,28], and verify the presence of conserved components in the venom of different species [29]. The production of toxin-specific antibodies could be the basis for a new generation of antivenoms capable of neutralizing clinically relevant toxins with greater efficiency. In this work, we compared these two approaches to determine which would be most viable to produce antivenoms against *B. arietans* venom for human use in sub-Saharan Africa.

## 2. Results

### 2.1. Profile of B. arietans Venom Obtained by Molecular Size Exclusion Chromatography

**Figure 1:**
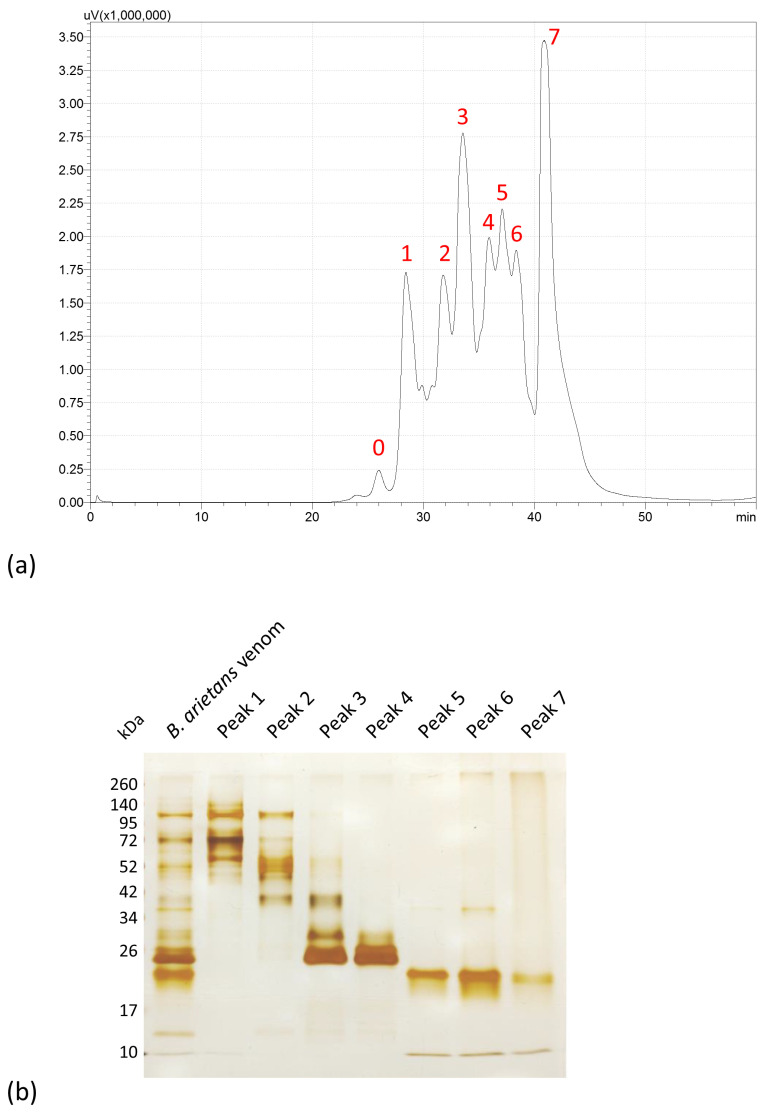
Profile of *B. arietans* venom obtained by molecular size exclusion chromatography and electrophoretic profiling. (**a**) Venom samples (100 µg) were fractioned in a Shim-Pack DIOL-300 column at a flow of 0.5 mL/min of disodium phosphate buffer. Absorbance was measured at 280 nm. (**b**) Fractions corresponding to crude venom (1 µg) and peaks 1 to 7 (2 µg) were submitted to 12.5% SDS-PAGE in non-reducing conditions and silver stained.

Fractionation of *Bitis arietans* venom was performed by molecular size exclusion chromatography. Eight individual peaks were recovered, labeled 0 to 7 (Figure 1a). Peaks 1 to 7 were submitted to dialysis, concentrated by filtering through Amicon filters (3 kDa), and had their protein content determined by the bicinchoninic acid (BCA) method using the commercial Pierce BCA Protein Assay kit (Rockford, IL, USA) (Table 1). The electrophoretic profile (Figure 1b) reveals the presence of higher molecular mass bands in peaks 1 and 2, with molecular masses at 95, 72, and 52 kDa. Peak 2 also shows a lighter molecular mass band, between 34 and 42 kDa. Peaks 3 and 4 show strongly marked bands with molecular masses close to 26 kDa. Additionally, peak 3 shows a band of greater molecular mass, between 34 and 42 kDa. Peaks 5 and 6 had a lightly marked band at 34 kDa, a band between 17 and 26 kDa, and a lightly marked band with a molecular mass under 10 kDa. Peak 7 shows bands with molecular mass similar to peaks 5 and 6, but less intense.

### 2.2. Recognition of Obtained Fractions by Western Blot and Immunoreactivity of Anti-Bitis antivenoms

**Figure 2:**
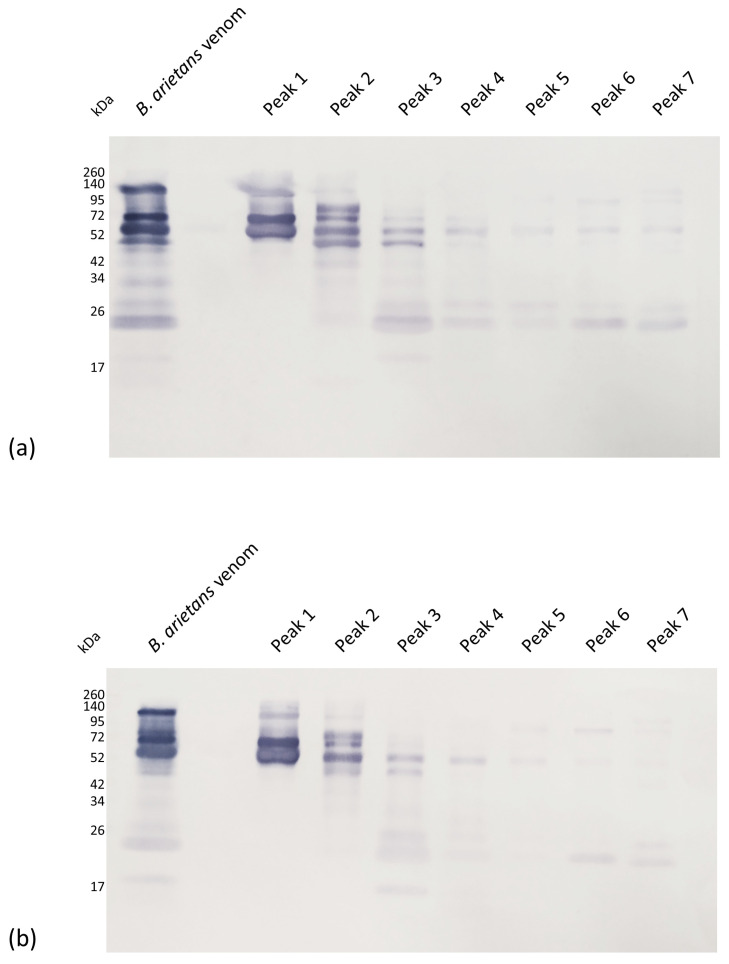
Western blot analysis showing the recognition of obtained peaks from *B. arietans* venom after size exclusion chromatography with monovalent anti-*B. arietans* (**a**) and polyvalent anti-*B. nasicornis* + *B. rhinoceros* antivenoms (**b**). *B. arietans* venom (8 µg) or peaks (3 µg) samples were submitted to 12.5% SDS-PAGE in non-reducing conditions under a current of 100 V and transferred to nitrocellulose membranes. Primary antibodies were diluted 1:1000 in PBS containing 0.1% BSA. The detection antibody (anti-horse conjugated with alkaline phosphatase) was diluted 1:7500 in PBS containing 0.1% BSA. Antibodies were incubated for 1 h at room temperature.

Recognition by monovalent anti-*Bitis arietans* antivenom (Figure 2a) reveals the presence of a wide range of bands present in the crude venom: 17 kDa, below 26 kDa, 26 kDa, 34 kDa, below 54 kDa, 54 kDa, 72 kDa, and 140 kDa. Bands of 54 kDa, 72 kDa, and 140 kDa could be seen in peak 1. Bands below 54 kDa, 54 kDa, and 72 kDa were identified in peak 2. Peaks 3, 4, 5, 6, and 7 show a very similar recognition profile, with bands between 17 and 26 kDa and bands close to 52 kDa. Recognition by polyvalent anti-*B. nasicornis* + *B. rhinoceros* antivenom (Figure 2b) reveals the presence of bands of 17 kDa, between 17 and 26 kDa, below 52 kDa, close to 52 kDa, close to 72 kDa, and 140 kDa were recognized in *B. arietans* venom. We can observe the recognition of bands in peak 1 (52, 72, and 140 kDa), peak 2 (close to 52 and 72 kDa), peak 3 (close to 17 kDa, between 17 and 26 kDa, and two bands close to 52 kDa), and peaks 4, 5, 6, and 7 (between 17 and 26 kDa and 52 kDa).

ELISA titration shows that both anti-*Bitis* antivenoms recognized proteins in all the peaks, but the titers obtained with the monovalent anti-*B. arietans* were slightly higher than the titers obtained with the polyvalent anti-*B. nasicornis* + *B. rhinoceros*. Peaks 1, 2, and 3 yielded the highest titers (Table 2).

### 2.3. Proteolytic Activity of Obtained Fractions and Differential Inhibition Assays

The proteolytic activity of fractions 1 to 6 and *B. arietans* venom was evaluated by hydrolysis of the FRET Abz-FRSSRQ-EDDnp substrate (Table 3). Peak 7 was not tested, as it was believed to be a mixture of many low molecular weight proteins and venom degradation products formed during the purification procedures. Peak 2 reveals the highest enzymatic activity, followed by peak 3, and then peaks 4, 5, and 6. Peak 1 did not show enzymatic activity in the substrate used. Based on the observed activity of peaks 2, 3, 4, and 5, they were selected for the next experimental steps.

Peaks 2, 3, 4, and 5 were submitted to the enzymatic inhibition assay using the substrate Abz-FRSSR-EDDnp with two differential inhibitors: EDTA (100 mM), a metalloprotease inhibitor, and PMSF (2 mM), an inhibitor of serine proteases. The samples were pre-incubated for 30 min with each of the inhibitors and then placed to react with the substrate. The reaction was monitored for 15 min, with readings every 30 s. Samples incubated with PBS were used as controls. Peaks 2, 3, and 4 were inhibited by PMSF but not by EDTA, indicating the presence of serine proteases. Peak 5 was inhibited only by EDTA, indicating the presence of metalloproteases (Table 4).

### 2.4. Identification of Peak Content by Mass Spectrometry

Mass spectrometry analysis of selected protein bands from peaks 2, 3, and 5 reveals the presence of lectins in all analyzed peaks. Serine proteases were identified in peaks 2 and 3, corroborating the result found in the enzymatic inhibition assay with PMSF. Only peak 5 shows the presence of disintegrins, non-enzymatic domains present in class II and III metalloproteases. The identification of a disintegrin, together with the result of enzymatic inhibition by EDTA, indicates the presence of metalloproteases in peak 5. Simplified MS results are shown in Table 5. A detailed image of the protein bands that were selected and the complete table of identified fragments are present in the Supplementary Materials (Supplementary Figure S1 and Supplementary Table S1). Our results indicate that, even with a simplified one-step chromatography protocol, we were able to obtain a high yield of immunogenic and clinically relevant toxins and, more importantly, the separation of serine proteases and metalloproteases in independent peaks. Peaks 2, 3, and 5 were selected for the immunization of BALB/c mice.

### 2.5. Monitoring of Immune Response Progression in Experimental Animals

**Figure 3:**
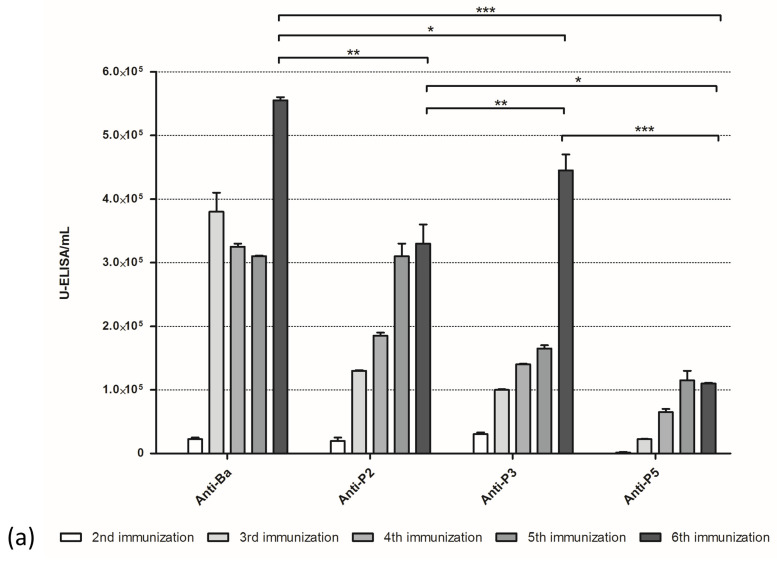

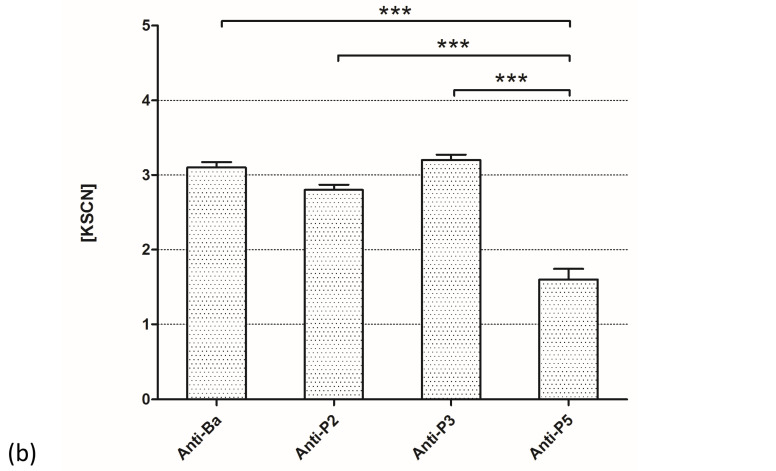
Progression of the immune response. (**a**) Balb/C mice (*n* = 7/group) were immunized with *B. arietans* venom (1.0 µg/animal) or with samples from peaks 2, 3, or 5 (0.5 µg/animal). Animals received 6 subcutaneous inoculations at intervals of 15 days. Before immunizations, blood was collected, and plasma was submitted to titration by ELISA. Plates were sensitized with 1 µg/well of *B. arietans* venom. Experimental antibodies were serially diluted from 1:1000 to 1:512,000 in PBS containing 0.1% BSA. Titers from the 6th immunization were compared between groups. (**b**) Samples from the 6th immunization were submitted for antibody affinity determination by ELISA. Antibodies were diluted 1:1000 in PBS containing 0.1% BSA. The affinity score was determined as the KSCN molarity necessary to displace 50% of the antibodies bound at KSCN 0 M. The detection antibody (anti-mouse IgG conjugated with peroxidase) was diluted 1:5000 in PBS containing 0.1% BSA. Experiments were performed in duplicate. Statistical analysis was performed by one-way ANOVA followed by Bonferroni’s post-test, * *p* < 0.05, ** p < 0.01, *** p < 0.001.

Antibody production was verified by titration of plasma samples recovered from every stage of the immunization schedule (Figure 3a). All experimental groups responded satisfactorily to immunization, with Anti-Ba (animals immunized with crude *B. arietans* venom) showing the highest titers, followed by Anti-P3 (animals immunized with peak 3) and Anti-P2 (animals immunized with peak 2). It is also possible to see that, in all groups, there was a marked increase in antibody production between the 2nd and 3rd immunizations. Anti-P5 (animals immunized with peak 5) shows lower titers compared to the other groups. Antibody affinity determination shows similar results from Anti-Ba, Anti-P2, and Anti-P3, with an antibody affinity index close to 3 M of KSCN. Once again, results from Anti-P5 were inferior, with an affinity index of 1.68 M for KSCN (Figure 3b). The consistently lower results observed with group Anti-P5 seem to indicate that the proteins present in peak 5 (metalloproteases) would be less immunogenic than the components present in peaks 2 and 3 (serine proteases), either because of size or some other intrinsic quality not determined.

### 2.6. Recognition Profile of Bitis spp. Venom by Experimental Antivenoms and Cross-Reaction Analysis

**Figure 4:**
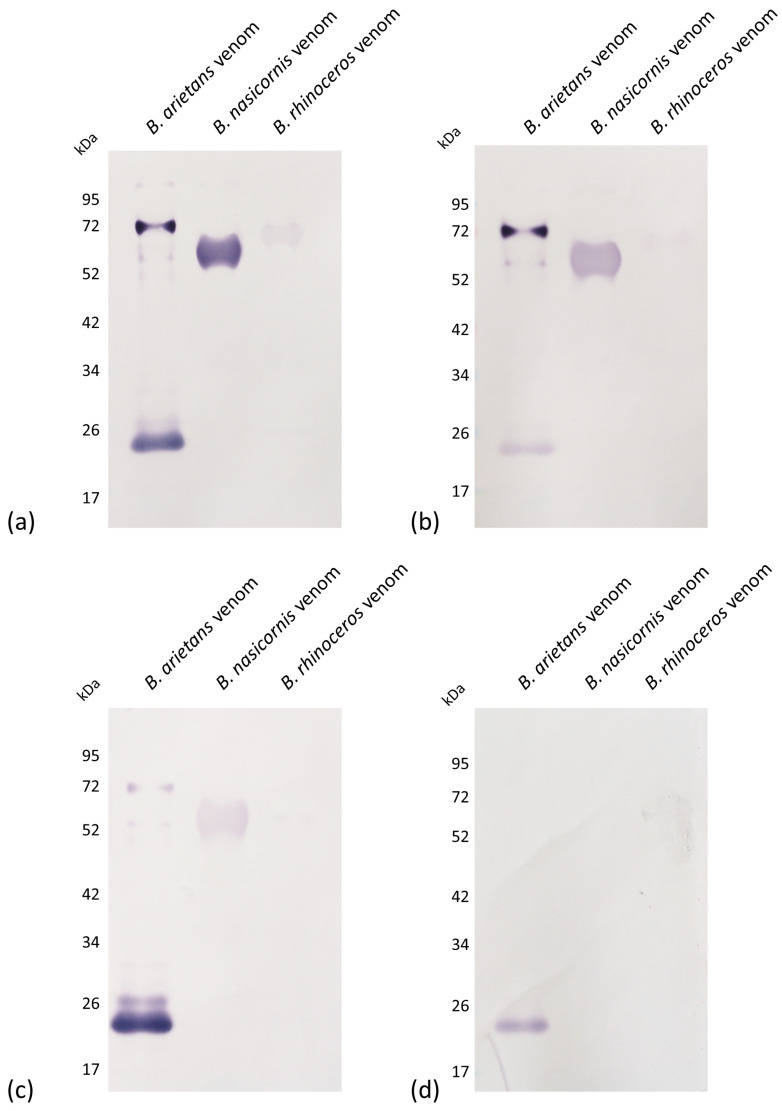
Recognition of protein bands present in *Bitis spp.* venoms by experimental antivenoms. Samples of *B. arietans*, *B. nasicornis*, and *B. rhinoceros* venom (5 µg) were submitted to 12.5% SDS-PAGE in non-reducing conditions and transferred to nitrocellulose membranes. Experimental antivenoms Anti-Ba (**a**), Anti-P2 (**b**), Anti-P3 (**c**), and Anti-P5 (**d**) were diluted 1:500 in PBS containing 0.1% BSA. The detection antibody (anti-mouse conjugated with alkaline phosphatase) was diluted 1:5000 in PBS containing 0.1% BSA. Antibodies were incubated for 1 h at room temperature.

Antivenoms from experimental groups Anti-Ba, Anti-P2, and Anti-P3 recognized the same bands present in *B. arietans* venom (24 kDa, 52 kDa, and 72 kDa), but with different degrees of intensity. Anti-P2 recognition was concentrated on bands of higher molecular mass, while Anti-P3 recognition was concentrated on the lighter molecular mass bands. This resemblance seems to indicate that the serine proteases, the components shared by the three groups, would be more immunogenic than the other proteins present in the whole venom and direct the immune response towards producing anti-SVSP antibodies. These three groups also recognized the same bands present in *B. nasicornis* venom (52 kDa) and *B. rhinoceros* venom (between 52 and 72 kDa), although with lower immunoreactivity. However, we observed that the cross-reaction with *B. nasicornis* venom was more intense. Anti-P5 only recognized a protein band close to 24 kDa in the *B. arietans* venom, and no bands were recognized in the other *Bitis* spp. venoms (Figure 4). 

**Figure 5:**
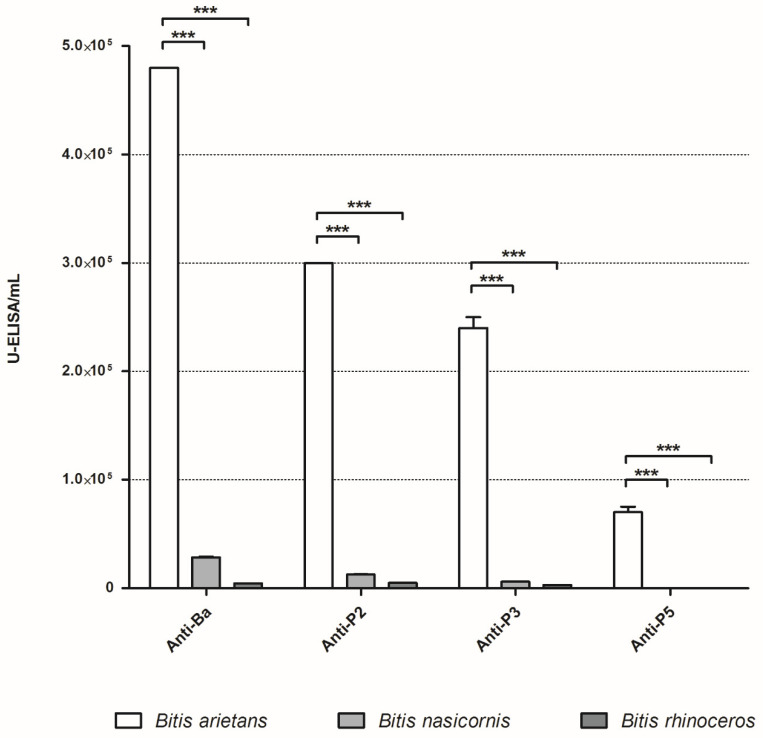
Cross-reaction with different *Bitis* spp. venoms. Plates were sensitized with 1 µg/well of antigen. Experimental antivenoms were serially diluted from 1:500 to 1:256,000 in PBS containing 0.1% BSA. Detection antibodies (anti-mouse IgG conjugated with peroxidase) were diluted 1:5000 in PBS containing 0.1% BSA. Absorbance at 420 nm was recorded. Titers are expressed as ELISA units/mL. Experiments were performed in duplicate. Statistical analysis was performed by one-way ANOVA followed by Dunnett’s post-test, *** p < 0.001.

Titration of experimental antivenoms against the *Bitis* spp. venoms shows a drastic difference in the recognition of the different venoms, with all experimental titers against *B. nasicornis* and *B. rhinoceros* being almost negligible (Figure 5).

### 2.7. In Vitro Inhibition of B. arietans Venom Activity with Experimental Antivenoms

The blocking potential of experimental antivenoms inhibition of *B. arietans* venom was determined by an inhibition assay using the FRET substrate Abz-FRSSRQ-EDDnp. All groups were able to partially inhibit substrate hydrolysis, although complete inhibition was not achieved. Once again, groups Anti-Ba, Anti-P2, and Anti-P3 show similar results. Group Anti-P5 had the lowest inhibitory action (Table 6). This indicates that the antivenoms were recognizing/neutralizing only a portion of the proteases present in the venom.

### 2.8. Protective Action of Experimental Antivenoms In Vivo

Experimental antivenoms were not able to prevent local hemorrhage induced by *B. arietans* venom in male Swiss mice. Measurement of the hemorrhagic halo of animals treated with venom + antivenom mixtures shows no decrease in area when compared to animals injected with crude venom (positive control) (Table 7), meaning inhibition was not observed. In fact, we noted an increase in halo size in animals treated with the experimental antivenoms, with groups Anti-P2 and Anti-P5 significantly larger than the positive control. Prevention of lethality induced by *B. arietans* venom was also not observed, and animals died within the first 6 h after injection. Necropsies reveal intense hemorrhage in the abdominal cavity. Hemorrhage is a consequence of the direct action of metalloproteases in the endothelium of blood vessels. Since our results had shown that groups Anti-Ba, Anti-P2, and Anti-P3 were directed towards SVSP recognition, we did not expect them to neutralize the metalloproteases. Group Anti-P5, although specific against SVMPs, only recognized low molecular weight proteins (below 24 kDa), and we believe that heavier and more hemorrhagic SVMPs (PII and PIII) were therefore neither recognized nor neutralized.

## 3. Discussion

Snake venoms are unique and complex mixtures of enzymatic and non-enzymatic components with different intrinsic characteristics, such as action mechanisms, immunogenicity, and relevance in the envenomation. In our understanding, the isolation and identification of key toxins could be a step towards developing a new generation of more specific and effective antivenoms. We determined that potential target toxins should be selected according to the following criteria: (1) be a major component of the whole venom; (2) be isolatable with relative ease; (3) be immunogenic; and (4) be clinically relevant in the context of human accidents. Using a single-step molecular exclusion chromatography protocol, we were able to obtain peaks containing enzymatically active serine proteases and metalloproteases from *B. arietans* venom, two of the most relevant toxins present in that venom. Animals were experimentally immunized with samples containing SVSPs, SVMPs, or crude *B. arietans* venom. Antibodies were recovered from plasma and subjected to an extensive battery of validation tests.

Our work has explored the limitations of both traditional and toxin-oriented approaches toward antivenom production. The main factor determining antibody production against a toxin is the immunogenicity of that protein. Immunogenicity, however, is not directly linked to the toxin’s relevance in human accidents, and even innocuous components can lead to the production of antibodies. In this study, we have demonstrated how the more immunogenic SVSPs directed the immune response of the experimental animals towards an anti-SVSP profile, even in animals immunized with crude venom. If this phenomenon was observed in animals immunized with a single snake venom, it is justifiable to question how the immune system of the serum-producing animals is responding to complex immunizing pools containing several different venoms. Guidolin et al. [30] observed that antivenom produced from animals immunized with a pool containing equal parts of *Naja annulifera*, *Dendroaspis angusticeps*, and *Dendroaspis polylepis* venoms was much more reactive against the *N. annulifera* venom and even other *Naja* spp. venoms than the *Dendroaspis* spp. venoms present in the pool. The presence of immunodominant venom components has been shown in other snake venoms. Kuniyoshi et al. [31] demonstrated that the commercial anti-Bothropic antivenom, produced by the Butantan Institute using an immunization pool composed of five different *Bothrops* spp. venoms, was very efficient in recognizing the bothropic SVMPs but did not react against the SVSPs, indicating a lack of specific antibodies against the serine proteases in the serum. Our results also show a very weak cross-reaction of the experimental antibodies with other *Bitis* spp. venoms. The taxonomy of the *Bitis* genus has been explored in depth by Calvete et al. [14], who demonstrated that *B. arietans* venom had unique characteristics when compared to the more closely related *B. nasicornis* and *B. rhinoceros*. This means that a broad-range anti-*Bitis* antivenom, developed by the traditional method of immunizing animals with a mixture of *B. arietans*, *B. nasicornis*, and *B. rhinoceros* venoms, would result in an antivenom with a very low specificity against each of those venoms, meaning that more ampules would have to be used in the treatment, increasing medical costs and the risk of adverse reactions.

The obtained antibodies, as experimentally indicated, were able to partially inhibit the crude venom enzymatic action on a specific substrate in vitro, but we did not observe in vivo protection. The observation that the developed antibodies anti-Ba, anti-P2, and anti-P3 were more directed towards the recognition of SVSPs could explain these antivenoms inability to prevent local hemorrhage, mostly induced by SVMPs. It is a known phenomenon that antigen-antibody reactions that take place in the antigen excess zone will lead to the formation of soluble immunocomplexes [32], which possess highly pro-inflammatory properties [33]. We believe this to be an explanation for the increase in hemorrhagic halo area in animals treated with the experimental antivenoms. The in vivo protocol used is also highly sensitive due to allowing for immediate venom and tissue contact. To overcome this, a dose-hemorrhage curve should have been made before the antibody neutralization assay. The lethality of a venom is the result of different toxins acting synergistically, and therefore, it is highly unlikely that the neutralization of a single toxin could prevent death. Still, the production of anti-toxin antibodies offers the possibility of a greater understanding of how snake venoms work, and the isolation and neutralization of specific toxins have been the focus of this and other studies. Megale et al. [34] isolated and characterized a fibrinogenolytic and kinin-releasing SVSP directly related to the hypotension observed in envenomation patients. Kuniyoshi et al. [31] were able to immunize animals with a pool of SVSPs isolated from Bothropic venom and develop specific anti-SVSP antibodies. Godoi et al. [35] isolated the less immunogenic SVMPs from the *B. arietans* venom and developed anti-SVMP antibodies capable of neutralizing the local hemorrhagic effects induced by that venom. These authors proposed that the toxin-specific antibodies, although not able to prevent lethality by themselves, could be used to enrich the commercial antivenoms, increasing their protection coverage. Despite being scientifically possible, this method is not viable for the large-scale production of antivenoms in the current model of production (immunization of large groups of horses). The complexity involved in having a separate group for each toxin family from every venom would exponentially increase the costs of production, defeating the purpose of creating an affordable antivenom. This approach, however, could be further explored at a laboratory scale on different antibody-generating platforms, such as monoclonal antibodies or phage-display libraries.

Although the traditional polyvalent antivenom production method works and is used worldwide to create commercial antivenoms, there are limitations that need to be addressed. Inoculating animals with large quantities of different venoms can lead to a final serum with an antibody profile skewed towards the more immunogenic components, independent of how relevant they are clinically. On the other hand, an antivenom designed to specifically neutralize a single toxin will only be able to partially neutralize the effects of the venom. With those limitations in mind, we would like to propose a different method for antivenom production: the development of polyclonal, species-specific, monovalent antivenoms. This approach would give us two points of fine-tuning over the traditional antivenom production process. The first is about the production of each monovalent antivenom itself. We can make punctual changes to the immunization procedures to adequately respond to each venom, therefore ensuring the proper generation of neutralizing antibodies. For example, less immunogenic toxins can be inoculated separately, different venoms can be paired with different adjuvants, and we can create different immunization schedules for each particular venom. The second point, and the crucial one, is the combination of the monovalent antivenoms to produce the polyvalent sera. By having control over this stage, we can customize the antivenom mixture to respect regional differences in herpetofauna. For example, an antivenom for use in countries where *B. arietans* is the only endemic species, like Namibia, Botswana, or Sudan, would contain only anti-*B. arietans* antibodies, whereas an antivenom designed for use in countries where all three main *Bitis* species are found, like Cameroon, Congo, or Gabon, would be composed of a mixture of one-third anti-*B. arietans*, one-third anti-*B. nasicornis*, and one-third anti-*B. rhinoceros* antibodies. By having an antivenom specifically formulated against the snakes present in a certain area, we reduce the quantity of irrelevant antibodies in each ampoule, therefore requiring fewer ampoules for the treatment, reducing costs, and, more importantly, preventing the occurrence of adverse reactions. This theoretical serum production model could be easily implemented, providing the African continent with a valuable agent against the snakebite problem.

## 4. Materials and Methods

### 4.1. Reagents

Disodium phosphate buffer (Na_2_HPO_4_, 200 mM; NaCl, 20 mM; pH 7), solution A for SDS buffer (Tris, 6.25 mM; SDS, 6.94 mM; pH 6.8), SDS buffer for non-reducing conditions (solution A, 9 mL; glycerol, 1 mL; bromophenol blue 1%, 2 mL), acrylamide 30% (acrylamide, 30 g; bis-acrylamide, 0.98 g; distilled H_2_O q.s. 100 mL), PBS buffer (KCl, 2.6 mM; KH_2_PO_4_, 1.5 mM; NaCl, 76 mM; Na_2_HPO_4_, 8.2 mM; pH 7.2), AP buffer (Tris HCl, 100 mM; NaCl, 100 mM; MgCl_2_, 5 mM; pH 9.5), NBT solution (NBT, 50 mg; dimethylphormamide, 700 µL; distilled H_2_O, 300 µL), BCIP solution (BCIP, 50 mg; dimethylphormamide, 1 mL), revealing solution for Western blot (AP buffer, 5 mL; NBT solution, 33 µL; BCIP solution, 16.5 µL), citrate buffer (citric acid, 0.1 M; NaH_2_PO_4_, 0.2 M; pH 5), OPD solution (OPD, 20 mg; citrate buffer, 1 mL), and substrate buffer for ELISA (citrate buffer, 5 mL; OPD solution, 100 µL; H_2_O_2_ 30 volumes, 5 µL).

### 4.2. Venoms

*Bitis arietans*, *Bitis nasicornis*, and *Bitis rhinoceros* venoms were supplied by Venom Supplies Ltd. (Tanunda, South Australia, Australia). According to the manufacturer, venoms were obtained from a population of snakes of both sexes with a medium length of 60 cm from the south of Africa. Lyophilized samples were stored at −20 °C. Venoms were diluted in PBS buffer, with their protein content assessed by the BCA method, and stored at −20 °C until use.

### 4.3. Fractionation of Proteins of Interest from B. arietans Venom

Fractions from *B. arietans* venom (batch BA33) were obtained by molecular exclusion chromatography using a Shim-Pack DIOL-300 column (7.9 mm × 50 cm) (Shimadzu Co., Kyoto, Japan) in an HPLC system (Shimadzu Co., Kyoto, Japan), previously equilibrated with disodium phosphate buffer, under a 0.5 mL/min flow of the same buffer. Samples were manually collected and oriented by absorbance at 280 nm with a UPC-900 ÄKTA HPLC monitor (GE Healthcare, Chicago, IL, USA). Samples were dialyzed in an Amicon Ultra system (3 kDa membrane, 5000× *g*, against 15 volumes of PBS buffer). Samples were stored at −20 °C.

### 4.4. Protein Quantification

Protein assessment of samples was performed by BCA [36] with a Pierce BCA Protein Assay Kit (Thermo Fisher Scientific Inc., Waltham, MA, USA), following the manufacturer’s instructions.

### 4.5. Electrophoretic Profile of B. arietans Venom and Isolated Fractions

Whole venom and its fractions were submitted to SDS-PAGE [37] (upper gel 5%, lower gel 12.5%), using a Mini-PROTEAN Tetra Cell system (Bio-Rad Laboratories, Hercules, CA, USA). Samples (2 µg) were diluted in SDS buffer under non-reducing conditions (1:1), heated for 6 min at 96 °C, applied in individual lanes, and separated at 100 V. Gels were either stained with silver sulfate [38] or electroblotted onto nitrocellulose membranes [39]. Molecular mass was estimated with Spectra™ Multicolor Broad Range Protein Ladder (Thermo Fisher Scientific Inc., Waltham, MA, USA).

### 4.6. Recognition of Protein Bands Present in B. arietans Venom and Fractions by Western Blot

Whole venom and its fractions were submitted to electrophoresis as described previously, and gel content was transferred to nitrocellulose membranes overnight at 4 °C at a current of 150 mA. Membranes were blocked with PBS containing 5% BSA for 2 h at 37 °C, washed with PBS, and treated with either equine anti-*B. arietans* or anti-*B. nasicornis + B. rhinoceros* antibodies, produced by Guidolin et al. [30], or the experimental murine antibodies, diluted to 1:1000 in PBS containing 0.1% BSA. Membranes were incubated for 1 h at room temperature and then washed three times with PBS containing 0.05% Tween 20. Afterwards, membranes were incubated with the detection antibody (anti-horse IgG (Sigma Aldrich, St. Louis, MO, USA), diluted to 1:7500 in PBS containing 0.1% BSA, or anti-mouse IgG (Sigma Aldrich, St. Louis, MO, USA), diluted to 1:5000 in PBS containing 0.1% BSA) for 1 h at room temperature, and then washed three times with PBS containing 0.05% Tween 20. Membranes were placed in a detection solution until staining was visualized, and the reaction was stopped by placing the membranes in distilled water.

### 4.7. Titration of Different Antivenoms against B. arietans Venom and Isolated Fractions

Antibody quantification was performed by ELISA. High-binding, 96-well plates were sensitized with 100 µL/well of whole venom or fractions (10 mg/mL in PBS) overnight at 4 °C. Following that, plates were blocked with 200 µL/well of PBS containing 5% BSA for 2 h at 37 °C. Plates were rinsed with 200 µL/well of PBS and incubated with 100 µL/well of the equine or murine antibodies (serially diluted from 1:1000 to 1:512,000 in PBS containing 0.1% BSA) for 1 h at 37 °C. Plates were washed three times with 200 µL/well of PBS containing 0.05% Tween 20 and incubated with 100 µL/well of the detection antibody (anti-horse IgG, diluted 1:20,000 in PBS containing 0.1% BSA, or anti-mouse IgG, diluted 1:5000 in PBS containing 0.1% BSA) for 1 h at 37 °C. Plates were washed three times with 200 µL/well of PBS containing 0.05% Tween 20, and we applied 50 µL/well of the substrate buffer. Plates were kept away from light for 15 min at room temperature. The reaction was stopped by the addition of 50 µL/well of H_2_SO_4_ (4 M). Absorbance at 490 nm was recorded using an ELISA plate reader (Labsystems Multiskan Ex, Thermo Scientific, Waltham, MA, USA). Titers were calculated in ELISA units/mL (one ELISA unit is defined by the antibody dilution, which results in an optical density of 0.2, as defined by Almeida et al. [40]).

### 4.8. Determination of Enzymatic Activity and Inhibition Essays of B. arietans Venom and Fractions

Enzymatic essays were performed according to the methodology described by Megale et al. [34], using 96-well opaque white plates. On one side of the well, 2 µL/well of FRET substrate Abz-FRSSRQ-EDDnp 250 mM were applied. On the other side, 2µg/well of whole venom or fractions were applied. The reaction was initiated by the addition of PBS (q.s. 100 µL/well). Hydrolyses of the substrate was monitored (excitation 320 nm, emission 420 nm) in a fluorescence spectrophotometer (Victor 3^TM^, Perkin-Elmer, Waltham, MA, USA) for 15 min with readings at every 30 s. For the selective inhibition essays, samples of whole venom and fractions were incubated for 30 min with PMSF 2 mM (serine protease inhibitor) or EDTA 100 mM (metalloprotease inhibitor) before being applied to the wells containing the substrate. Samples incubated with PBS were used as controls. To verify the inhibitory potential of the experimental antivenoms, we replaced PMSF and EDTA with antivenom samples (1:5 proportion of venom: antivenom), incubated for 30 min at 37 °C under continuous stirring.

### 4.9. Mass Spectrometry Analysis of Bands from B. arietans Venom

Samples of the peaks obtained by size exclusion chromatography (5 µg) were submitted to SDS-PAGE as previously described and proceeded with gel tryptic digestion (Sigma-Aldrich, St. Louis, MO, USA) [41]. Briefly, gel bands were excised from the gel, and gel pieces were de-stained two times with 50% methanol containing 5% acetic acid for 30 min each. Gel pieces were dehydrated using acetonitrile (can) for two cycles of 5 min and dried in a Speed-Vac vacuum concentrator (RVC 2-18, Christ, Osterode am Harz, Germany). Reduction was performed by incubating the samples with 10 mM dithiothreitol at room temperature for 30 min and then alkylated by incubating them with 50 mM iodoacetamide at room temperature in the dark for 30 min. After washing with 100 mM ammonium bicarbonate, gel pieces were dehydrated with 100% ACN for 5 min and rehydrated with 100 mM ammonium bicarbonate. Proteins in the processed gel pieces were digested with a freshly prepared trypsin solution containing 50 μg/mL of trypsin (Sigma-Aldrich, St. Louis, MO, USA) in 50 mM ammonium bicarbonate at 37 °C for 16 h, then extracted with 100% ACN containing 1% TFA. To assure clearance of all gel fragments, samples were cleaned up using C-18 ZipTip (Merck Millipore, Burlington, MA, USA). Finally, the extracted tryptic peptides were lyophilized and reconstituted with 5 μL of 0.1% formic acid. Samples were analyzed in an LTQ-Orbitrap Velos (Thermo Fisher Scientific, Bremen, Germany) coupled to an EASY 1000 nano-liquid chromatographer (Thermo Fisher Scientific, Bremen, Germany). The spectrometer was equipped with a nano electrospray ionizing source connected to an in-house-prepared 10 cm analytical column (75 µm I.D. × 350 µm O.D.) loaded with Jupiter^®^ C-18 5 µm beads (Phenomenex, Torrance, CA, USA). The precolumn (7 cm × ID 75 μm × OD 360 μm) was also prepared in house and packed with 5 cm of 10 μm C18 resin (Phenomenex, Torrance, CA, USA). Peptides were eluted in a linear gradient (200 nL/min flow) of acetonitrile 5–95% containing 0.1% formic acid for 15 min. MS spectra were acquired using a scan range of 200–2000 *m*/*z*; a full scan resolution of 60,000; a max time of injection of 100 ms; an electrospray voltage of 2.1 kV, and a source temperature of 200 °C. The mass spectrometer was operated in data-dependent acquisition (DDA) mode, with the 10 most intense peaks selected for CID fragmentation (isolation window of 2 Da; exclusion time 15 s; minimal signal 5000; activation time = 10 ms; normalized collision energy = 35%). The raw data (RAW) were analyzed using PEAKS Studio version 8.5 (Bioinformatics Solutions, Waterloo, ON, Canada) [42,43]. A false discovery rate (FDR) of 1% was required for both protein and peptide identifications. Spectra were searched against reviewed “*Bitis*” (317 entries) and “Serpents” (149.820 entries) sequences from the UNIPROT database (Taxid: 8570) (July 2019). Enzyme specificity was set to trypsin, and at least two missed cleavages were allowed; cysteine carbamidomethylation was selected as a fixed modification, while methionine oxidation, glutamine, or asparagine deamidation, and protein N-terminal acetylation were selected as variable modifications. Peptide identification was based on a search with a mass deviation of the precursor ion of 10 ppm and the fragment mass tolerance was set to 0.5 Da.

### 4.10. Immunization Procedures and Processing of Hyperimmune Plasmas

Whole *B. arietans* venom and peaks 2, 3, and 5 were selected for the immunization procedure. Female BALB/c mice were divided into 4 groups (*n* = 7/group) and injected with the venom (1.0 µg/animal) or with the peak of interest (0.5 µg/animal), with mesoporous silica as adjuvant (1:10 proportion of antigen: SBA) [44]. Animals received 6 subcutaneous inoculations of antigen + adjuvant in saline solution (NaCl 0.15 M) at intervals of 15 days. Blood was collected by submandibular plexus puncture, and 15 days after the 6th immunization, animals were submitted to anesthetic overdosage of xylazine (30 mg/kg) + ketamine (300 mg/kg). Blood was drawn by cardiac puncture. Blood samples were collected in heparin and centrifuged at 3000 RPM for 30 min. Plasmas were collected, heated at 56 °C for 30 min to inactivate complement, and pooled within their respective groups. Samples were stored at −20 °C until use.

### 4.11. Affinity Determination of Experimental Antivenoms to B. arietans Venom

Antivenom affinity was determined by ELISA, as described in 4.7, with two modifications. Experimental antivenoms had a fixed dilution of 1:1000, and the incubation with the experimental antibodies was immediately followed by an incubation step with KSCN, 100 µL/well (Sigma Aldrich, St. Louis, MO, USA) [45,46], in concentrations ranging from 0 to 5 M, in 1 M intervals, for 30 min at room temperature. After this, the reaction continued as described previously. The affinity index was calculated as the KSCN concentration needed to reduce by 50% the optical density obtained with the control (KSCN 0 M).

### 4.12. Inhibition of Local Hemorrhage Induced by B. arietans Venom

Male Swiss mice (18–20 g) were divided into six groups (*n* = 3/group) and intradermally inoculated with 50 µL/animal one of the following: PBS (negative control), 10 µg of *B. arietans* venom (positive control), or a mixture of 10 µg of venom + experimental antivenoms (1:10), previously incubated for 30 min at 37 °C under gentle stirring. After three hours, the animals were sacrificed by overdose of the anesthetic solution xylazine (30 mg/kg) + ketamine (300 mg/kg). The skin around the site of inoculation was surgically removed, and the hemorrhagic area was photographed and measured. The procedures described here were performed in accordance with Butantan Institute (9029281119) and the University of São Paulo Ethical Committees for Animal Experimentation (5710281119).

### 4.13. Serum Neutralization of Lethality Induced by B. arietans Venom

Male Swiss mice (18–20 g) were divided into groups (*n* = 4/group) and intraperitonially injected with 500 µL of one 100 µg of *B. arietans* venom (equivalent to 2 LD_50_) diluted in PBS or mixtures of *B. arietans* venom (2 LD_50_) + 1:5 or 1:10 dilutions of experimental antivenoms, previously incubated for 30 min at 37 °C under gentle stirring. Deaths were recorded at 0 h, 12 h, 24 h, and 48 h after injections. The effective dose (ED_50_) was determined by probits [47]. Procedures described here are in accordance with WHO standards [48], and the Butantan Institute (9029281119), and the University of São Paulo Ethical Committees for Animal Experimentation (5710281119).

### 4.14. Statistical Analysis

Obtained data were expressed as a medium ± standard deviation and analyzed by one-way ANOVA followed by Bonferroni’s or Dunnett’s tests, when applicable. A statistical difference was considered significant when *p* < 0.05. Analysis was performed with GraphPad Prism version 5.01 for Windows (GraphPad Software, San Diego, CA, USA).

## Figures and Tables

**Table 1:** Estimation of protein content of peaks obtained after fractionation of *B. arietans* venom by size exclusion chromatography.

Sample	Concentration (µg/mL)
Peak 1	436.26 ± 13.12
Peak 2	376.43 ± 14.92
Peak 3	1215.58 ± 38.32
Peak 4	259.55 ± 5.54
Peak 5	300.23 ± 14.82
Peak 6	196.97 ± 1.84
Peak 7	237.42 ± 1.84

Protein content quantification was performed with the Pierce BCA Protein Assay Kit (Thermo Fisher Scientific, Waltham, MA, USA), following the manufacturer’s instructions. Experiments were performed in duplicate.

**Table 2:** Titration of anti-*Bitis* antivenoms against the obtained peaks from *B. arietans* venom after size exclusion chromatography.

Sample	Anti-*B. arietans*	Anti-*B. nasicornis + B. rhinoceros*
*B. arietans* venom	(4.10 ± 0.01) × 10^5^	(2.80 ± 0.20) × 10^5^
Peak 1	(1.80 ± 0.01) × 10^5^	(1.85 ± 0.05) × 10^5^
Peak 2	(3.15 ± 0.05) × 10^5^	(2.05 ± 0.05) × 10^5^
Peak 3	(2.20 ± 0.01) × 10^5^	(1.15 ± 0.05) × 10^5^
Peak 4	(0.75 ± 0.01) × 10^5^	(0.60 ± 0.05) × 10^5^
Peak 5	(0.86 ± 0.01) × 10^5^	(0.50 ± 0.01) × 10^5^
Peak 6	(0.92 ± 0.02) × 10^5^	(0.62 ± 0.02) × 10^5^
Peak 7	(0.44 ± 0.01) × 10^5^	(0.40 ± 0.01) × 10^5^

High-binding, 96-well plates were sensitized with 1 µg/well of antigen. Primary antibodies were serially diluted in the interval of 1:1000 to 1:512,000 in PBS containing 0.1% BSA. Detection antibodies (anti-horse IgG conjugated with peroxidase) were diluted 1:20,000 in PBS containing 0.1% BSA. Absorbance at 420 nm was recorded. Titers are expressed as ELISA units/mL.

**Table 3:** Proteolytic activity of *B. arietans* venom and isolated fractions on Abz-FRSSRQ-EDDnp substrate.

Sample	Enzymatic Activity (∆A × min^−1^ × µg^−1^)
*B. arietans* venom	2544.45 ± 76.43
Peak 1	54.62 ± 9.38
Peak 2	3401.77 ± 100.10
Peak 3	364.60 ± 1.27
Peak 4	172.88 ± 32.72
Peak 5	170.77 ± 2.50
Peak 6	125.83 ± 21.03

Samples of venom (1 µg/well), peaks (2 µg/well), and the FRET substrate (5 µM/well) were applied in 96-well white-opaque plates, and the reaction was initiated by the addition of PBS (q.s. 100 µL). The reaction was monitored in a fluorescence spectrofluorometer (excitation 320 nm, emission 420 nm) for 15 min. UF = fluorescence units. Experiments were performed in duplicate. Enzymatic activity is expressed as ∆ absorbance per minute per µg of toxin.

**Table 4:** Inhibition assays of selected peaks with differential inhibitors.

Sample	Treatment	Enzymatic Activity (∆A × min^−1^ × µg^−1^)
Peak 2	PBS (control)	3061.50 ± 105.17
	PMSF	435.97 ± 161.43 ***
	EDTA	2544.17 ± 259.57
Peak 3	PBS (control)	394.58 ± 35.75
	PMSF	29.62 ± 5.22 ***
	EDTA	423.22 ± 43.12
Peak 4	PBS (control)	184.87 ± 8.47
	PMSF	20.57 ± 0.57 ***
	EDTA	170.17 ± 1.00
Peak 5	PBS (control)	184.62 ± 5.38
	PMSF	204.92 ± 24.92
	EDTA	49.02 ± 0.92 ***

Samples of (a) peak 2 (1 µg/well), (b) peak 3 (2 µg/well), (c) peak 4 (2 µg/well), and (d) peak 5 (2 µg/well) were incubated with PMSF (2 mM) or EDTA (100 mM) for 30 min and applied to the FRET substrate Abz-FRSSRQ-EDDnp (5 µM). Samples incubated with PBS were used for comparison. The reaction was monitored in a fluorescence spectrophotometer, Hidex Sense (Hidex, Finland) (λEX 320 nm, λEM 420 nm), for 15 min. UF = fluorescence units. Experiments were performed in duplicate. Enzymatic activity is expressed as ∆ absorbance per minute per µg of toxin. Statistical analysis was performed by one-way ANOVA followed by Dunnett’s post-test, *** *p* < 0.001.

**Table 5:** Identification of protein band content by LC-MS/MS from fractions obtained from *B. arietans* venom after size exclusion chromatography.

Sample	Major Families of Identified Peptides
Peak 2	Snaclecs, C-type lectins, serine proteases
Peak 3	Snaclecs, C-type lectins, serine proteases
Peak 5	C-type lectins, disintegrins

FG1 to FG6 were cut from the gel and subjected to in-gel digestion with trypsin. Analysis was performed using an Easy-nLC Proxeon nanoHPLC system coupled to an LTQ-Orbitrap Velos. The results obtained were compared with the “*Bitis*” and “Snakes” databases downloaded from Uniprot (Taxid: 8570).

**Table 6:** Inhibitory role of experimental antivenoms on *B. arietans* venom activity.

Sample	Enzymatic Activity (∆A × min^−1^ × µg^−1^)
Venom + PBS (control)	1790.37 ± 70.70
Venom + Anti-Ba	453.70 ± 47.83 ***
Venom + Anti-P2	354.47 ± 3.33 ***
Venom + Anti-P3	487.17 ± 5.50 ***
Venom + Anti-P5	763.83 ± 19.97 ***

*B. arietans* venom samples (1 µg) were incubated with experimental antivenoms (1:5) for 30 min and applied to the FRET substrate Abz-FRSSRQ-EDDnp (5 µM). The reaction was monitored in a fluorescence spectrophotometer (λEX 320 nm, λEM 420 nm) for 15 min. UF = fluorescence units. Samples incubated with PBS were used for comparison. Experiments were performed in duplicate. Enzymatic activity is expressed as ∆ absorbance per minute per µg of toxin. Statistical analysis was performed by one-way ANOVA followed by Dunnett’s post-test, *** p < 0.001.

**Table 7:** Measurement of hemorrhagic halos induced by *B. arietans* venom and venom + experimental antivenom injection.

Treatment	Hemorrhagic Area	Inhibition
Negative control (PBS)	0 mm^2^	-
Positive control (venom)	47 ± 6 mm^2^	-
Anti-Ba	63 ± 6 mm^2^	no
Anti-P2	73 ± 9 mm^2^ **	no
Anti-P3	50 ± 7 mm^2^	no
Anti-P5	67 ± 16 mm^2^ *	no

Male Swiss mice (*n* = 4/group) were intradermally inoculated in the dorsal region with preparations of 10 µg/animal of venom + experimental antivenoms (1:10), previously incubated for 30 min at 37 °C under gentle stirring. After 3 h, the animals were sacrificed, and the skin was surgically removed. Negative control: animals inoculated with PBS; positive control: animals inoculated with 10 µg/animal of venom. Inhibition was considered if any decrease in area compared to the positive control (venom) was observed. Statistical analysis was performed by one-way ANOVA followed by Dunnett’s post-test, * *p* < 0.05, ** p < 0.01.

## Data Availability

The data obtained in this research are available for consultation through the email address guidolin.felipe@gmail.com. Data is not available on other sites for consultation.

## Supplementary Materials

The following supporting information can be downloaded at: https://www.mdpi.com/article/10.3390/toxins15090584/s1.
