# Supplementary material for: Comparing Traditional and Toxin-Oriented Approaches towards Antivenom Production against Bitis arietans Snake Venom
Source: Toxins (Basel). 2023 Sep 20;15(9):584. doi: 10.3390/toxins15090584 (PMC10537286; doi:10.3390/toxins15090584)
Supplement: Supplementary file 1 [file toxins-15-00584-s001.zip › toxins-2563270-supplementary.pdf]

# Comparing Traditional and Toxin-Oriented Approaches towards Antivenom Production against *Bitis arietans* Snake Venom

**Felipe Raimondi Guidolin [1,*], Kemily Stephanie de Godoi [1], Angela Alice Amadeu Megale [1], Cristiane Castilho Fernandes da Silva [2], Roberto Tadashi Kodama [2], Daniela Cajado-Carvalho [3], Leo Kei Iwai [3], Patrick Jack Spencer [4], Fernanda Calheta Vieira Portaro [2] and Wilmar Dias da Silva [1,*]**

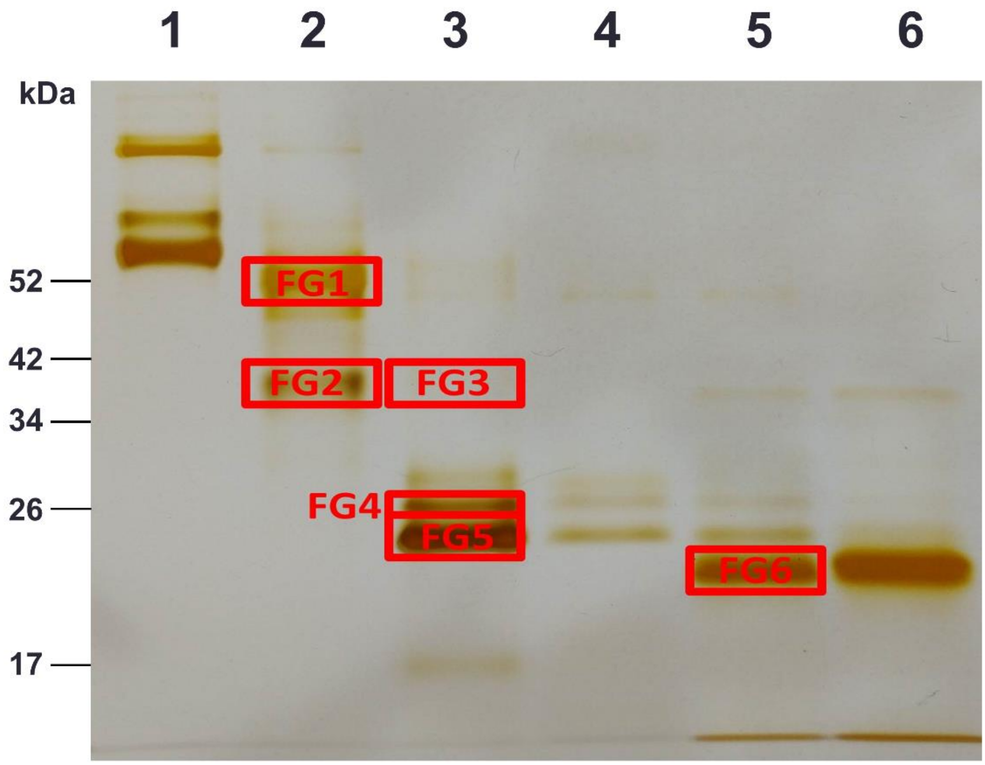


**Supplementary Figure S1. Selection of protein bands from peaks 2, 3 and 5 from fractionating of *B. arietans* venom by size exclusion chromatography to protein identification on LC-MS/MS analysis.** 5 µg of each peak were submitted to 12.5% SDS-PAGE, in non-reducing conditions. Selected bands (FG1 to FG6) were cut and submitted to in-gel trypsin digestion.

**Supplementary Table S1. Identification of protein bands content by LC-MS/MS from fractions obtained from *B. arietans* venom af ter size exclusion chromatography.**

| Sample | Score | Accession | Description | OS |
|---|---|---|---|---|
| FG1 | 78 | SLRB_BITHR | Snaclec rhinocetin subunit beta | *Bitis rhinoceros* |
| | 49 | A0A140DC05_BITAR | C-type lectin 1b | *Bitis arietans* |

| | | | | |
|---|---|---|---|---|
| | **50** | **SLA_BITAR** | **Snaclec bitiscetin subunit alpha** | ***Bitis arietans*** |
| | **32** | **A0A0B6VJU7_PROFL** | **Beta-actin** | ***Protobothrops flavoviridis*** |
| | **32** | **A0A0F7Z857_CROAD** | **Poly(a)-specific ribonuclease PARN** | ***Crotalus adamanteus*** |
| | **28** | **A0A1I9KNN4_VIPAA** | **Serine proteinase SP-3** | ***Vipera ammodytes ammodytes*** |
| | **28** | **A0A0F7Z8B1_CROAD** | **E3 ubiquitin-protein ligase** | ***Crotalus adamanteus*** |
| | **18** | **A0A6B2FD68_9SAUR** | **C-type lectin domain-containing protein** | ***Bothriechis nubestris*** |
| | **17** | **A0A0K8RXM4_CROHD** | **Cyclin-dependent kinase 1-like** | ***Crotalus horridus*** |
| | **131** | **A0A194AM96_9SAUR** | **Serine proteinase 17** | ***Agkistrodon piscivorus*** |
| | **42** | **A0A194APC3_9SAUR** | **Serine proteinase 14b** | ***Agkistrodon piscivorus*** |
| | **58** | **A0A8A8C7M3_BOTJA** | **Thrombin-like enzyme** | ***Bothrops jararaca*** |
| | **56** | **VSPY_MACLB** | **Chymotrypsin-like protease VLCTLP** | ***Macrovipera lebetina*** |
| | **66** | **E9JG14_ECHCS** | **Serine protease (fragment)** | ***Echis carinatus sochureki*** |
| | **61** | **D5KRX9_ECHOC** | **Snake venom serine protease (fragment)** | ***Echis ocellatus*** |
| | **116** | **A0A6B7FPJ0_VIPAA** | **Serine proteinase SP-8** | ***Vipera ammodytes ammodytes*** |
| | **81** | **J3RZT5_CROAD** | **Keratin, type I cytoskeletal 19** | ***Crotalus adamanteus*** |
| | **58** | **SLRB_BITRH** | **Snaclec rhinocetin subunit beta** | ***Bitis rhinoceros*** |
| | **50** | **SLA_BITAR** | **Snaclec bitiscetin subunit alpha** | ***Bitis arietans*** |
| | **57** | **A0A2H4Z2W1_DABSI** | **RVV-X heavy chain** | ***Dabola siamensis*** |
| | **52** | **A0A0F7ZB82_CROAD** | **Ankyrin repeat and BTB/POZ domain-containing protein 2** | ***Crotalus adamanteus*** |
| | **48** | **A0A1I9KNL5_VIPAA** | **Serine proteinase SP-2** | ***Vipera ammodytes ammodytes*** |

| | | | | |
|---|---|---|---|---|
| FG2 | 45 | A0A0B8RV71_CROHD | Keratin, type II cytoskeletal cochleal-like | *Crotalus horridus* |
| | 44 | OXLA_DABRR | L-amino-acid oxidase | *Daboia russelii* |
| | 43 | A0A0F7ZAB1_CROAD | Keratin, type I cytoskeletal 18-like protein | *Crotalus adamanteus* |
| | 36 | A0A1W7RD94_AGKCO | Sister chromatid cohesion protein PDS5 | *Agkistrodon contortrix contortrix* |
| | 34 | A0A0F7ZBJ1_CROAD | FAS-associated factor 1 | *Crotalus adamanteus* |
| | 33 | A0A1W7REY6_AGKCO | Sister chromatid cohesion protein PDS5 B | *Agkistrodon contortrix contortrix* |
| | 33 | A0A0F7Z8R3_CROAD | Interferon-inducible GTPase 5-like | *Crotalus adamanteus* |
| | 32 | A0A0F7Z8B1_CROAD | E3 ubiquitin-protein ligase | *Crotalus adamanteus* |
| | 29 | A0A194APM7_9SAUR | C-type lectin 9d | *Agkistrodon piscivorus* |
| | 28 | A0A0F7ZEI3_CROAD | Protein c20orf29 | *Crotalus adamanteus* |
| | 27 | A0A0F7Z857_CROAD | Poly(A)-specific ribonuclease PARN | *Crotalus adamanteus* |
| | 26 | A0A0F7ZE88_CROAD | Dermatan-sulfate epimerase | *Crotalus adamanteus* |
| | 26 | A0A1W7RF54_AGKCO | NudC domain-containing protein 2 | *Agkistrodon contortrix contortrix* |
| | 25 | A0A0F7Z7G6_CROAD | PAT1 1-like protein | *Crotalus adamanteus* |
| | 23 | J3SFD0_CROAD | SURF1-like protein | *Crotalus adamanteus* |
| | 20 | A0A0F7Z8B2_CROAD | Exosome component 10 isoform 1 | *Crotalus adamanteus* |
| | 19 | A0A0F7Z5N5_CROAD | Polycomb protein SCMH1 isoform X2 | *Crotalus adamanteus* |
| | 19 | A0A0F7ZCD4_CROAD | Oxidation resistance protein 1 isoform X3 | *Crotalus adamanteus* |
| | 19 | A0A0F7Z415_CROAD | Serine/threonine-protein kinase TBK1-like protein | *Crotalus adamanteus* |
| | 18 | A0A0F7ZEN3_CROAD | Ankyrin repeat domain-containing protein 12-like | *Crotalus adamanteus* |

| | | | | |
|---|---|---|---|---|
| | 16 | A0A0F7Z988_CROAD | Creatine kinase | *Crotalus adamanteus* |
| | 16 | A0A1W7RHL5_AGKCO | Endoplasmic reticulum-Golgi intermediate compartment protein 2-like protein | *Agkistrodon contortrix contortrix* |
| | 14 | A0A0F7Z6K8_CROAD | Dynamin_N domain-containing protein | *Crotalus adamanteus* |
| | 13 | T1E5M6_CROHD | Acetyl-CoA C_myristoyltransferase | *Crotalus horridus* |
| FG3 | 73 | VSPC_TRIGA | Snake venom serine protease 2C | *Trimeresurus gramineus* |
| | 68 | J3RZT5_CROAD | Keratin, type I cytoskeletal 19 | *Crotalus adamanteus* |
| | 61 | A0A194AM96_9SAUR | Serine proteinase 17 | *Agkistrodon piscivorus* |
| | 54 | D5KRX9_ECHOC | Snake venom serine protease (fragment) | *Echis ocellatus* |
| | 52 | D8MIA2_BITRH | Snake venom serine protease (fragment) | *Bitis rhinoceros* |
| | 47 | A0A6B7FPJ0_VIPAA | Serine proteinase SP-8 | *Vipera ammodytes ammodytes* |
| | 42 | A0A194APM7_SAUR | C-type lectin 9d | *Agkistrodon piscivorus* |
| | 33 | A0A0F7ZAB1_CROAD | Keratin, type I cytoskeletal 18-like protein | *Crotalus adamanteus* |
| | 29 | A0A5A4WNG2_BITAR | Bitiscetin-3 subunit alpha | *Bitis arietans* |
| | 24 | A0A1W7RC08_AGKCO | Ubiquitin-like conjugating enzyme ATG3-like protein | *Agkistrodon contortrix contortrix* |
| | 23 | A0A0K8S0L2_CROHD | Iron-sulfur cluster assembly 1, mitochondrial-like protein | *Crotalus horridus* |
| | 23 | A0A0F7Z8B1_CROAD | E3 ubiquitin-protein ligase | *Crotalus adamanteus* |
| | 22 | A0A0F7ZEI3_CROAD | Protein C20orf29 | *Crotalus adamanteus* |
| | 22 | A0A0F7Z7A4_CROAD | Protein phosphatase 1 regulatory subunit 12B-like | *Crotalus adamanteus* |
| | 22 | A0A1W7RF54_AGKCO | NudC domain-containing protein 2 | *Agkistrodon contortrix contortrix* |

| | | | | |
|---|---|---|---|---|
| | **21** | **A0A0F7ZBJ1_CROAD** | **FAS-associated factor 1** | ***Crotalus adamanteus*** |
| | **21** | **A0A0F7Z958_CROAD** | **Guanine nucleotide-binding protein subunit alpha-11** | ***Crotalus adamanteus*** |
| | **20** | **A0A0K8RWZ3_CROHD** | **Proteasome-associated protein ECM29-like** | ***Crotalus horridus*** |
| | **19** | **A0A0F7Z3D0_CROAD** | **Ubiquitin C** | ***Crotalus adamanteus*** |
| | **19** | **A0A0F7Z434_CROAD** | **RAD21-like protein** | ***Crotalus adamanteus*** |
| | **19** | **A0A2H4Z2W1_DABSI** | **RVV-X heavy chain** | ***Daboia siamensis*** |
| | **18** | **A0A6B2FB67_BOTNI** | **U6 snRNA-associated Sm-like protein LSm8** | ***Bothriechis nigroviridis*** |
| **FG4** | **37** | **I7JA31_BITRH** | **C-type lectin-like protein 4** | ***Bitis rhinoceros*** |
| | **33** | **A0A0C5DKL1_MACLB** | **C-type lectin-like protein 3A** | ***Macrovipera lebetina*** |
| | **33** | **A0A140DC06_BITAR** | **C-type lectin 2** | ***Bitis arietans*** |
| | **33** | **A0A1B3AXS3_BITAR** | **C-type lectin-like protein** | ***Bitis arietans*** |
| | **33** | **A0A6B7FMP6_VIPAA** | **C-type lectin-like Snaclec-9** | ***Vipera ammodytes ammodytes*** |
| | **33** | **SLAE_MACLB** | **Snaclec A-14** | ***Macrovipera lebetina*** |
| | **33** | **SLAF_MACLB** | **Snaclec a15** | ***Macrovipera lebetina*** |
| | **33** | **SLA_DABPA** | **Snaclec VP12** | ***Daboia palaestinae*** |
| | **33** | **SLA2_BITGA** | **Snaclec 2** | ***Bitis gabonica*** |
| | **33** | **I7JX23_BITRH** | **C-type lectin-like protein 3** | ***Bitis rhinoceros*** |
| | **33** | **K9JBV0_DABSI** | **P68 alpha subunit** | ***Daboia siamensis*** |
| | **33** | **K9JDF2_DABRR** | **P68 alpha subnunit** | ***Daboia russelii limitis*** |
| | **27** | **A0A0C5DKK6_MACLB** | **C-type lectin-like protein 2A** | ***Macrovipera lebetina*** |
| | **27** | **SLMA_MACLB** | **Snaclec macrovipecetin subunit alpha** | ***Macrovipera lebetina*** |

| | | | | |
|---|---|---|---|---|
| FG5 | 54 | SLA_BITAR | Snaclec bitiscetin subunit alpha | *Bitis arietans* |
| | 52 | SLB6_MACLB | Snaclec B6 (fragment) | *Macrovipera lebetina* |
| | 49 | A0A0C5DKL1_MACLB | C-type lectin-like protein 3A | *Macrovipera lebetina* |
| | 46 | J3RZT5_CROAD | Keratin, type I cytoskeletal 19 | *Crotalus adamanteus* |
| | 40 | A0A1W7RE48_AGKCO | Ribonuclease P protein subunit p38-like protein | *Agkistrodon contortrix contortrix* |
| | 38 | A0A0B8RV71_CROHD | Keratin, type II cytoskeletal cochleal-like | *Crotalus horridus* |
| | 34 | A0A0F7ZAB1_CROAD | Keratin, type I cytoskeletal 18-like protein | *Crotalus adamanteus* |
| | 31 | A0A0F7Z805_CROAD | MBT domain-containing protein 1 isoform | *Crotalus adamanteus* |
| | 29 | SLB1_MACLB | Snaclec B1 | *Macrovipera lebetina* |
| | 29 | A0A0F7Z8B1_CROAD | E3 ubiquitin-protein ligase | *Crotalus adamanteus* |
| | 29 | A0A194APM7_9SAUR | C-type lectin 9d | *Agkistrodon piscivorus* |
| | 28 | A0A1W7RD94_AGKCO | Sister chromatid cohesion protein PDS5 | *Agkistrodon contortrix contortrix* |
| | 26 | A0A1W7REY6_AGKCO | Sister chromatid cohesion protein PDS5 B | *Agkistrodon contortrix contortrix* |
| | 26 | SL114_ECHCA | Snaclec carinactivase-1 regulatory subunit 14 kDa chain (fragment) | *Echis carinatus* |
| | 25 | A0A0C5DKK6_MACBL | C-type lectin-like protein 2A | *Macrovipera lebetina* |
| | 24 | A0A1W7RC08_AGKCO | Ubiquitin-like-conjugating enzyme ATG3-like protein | *Agkistrodon contortrix contortrix* |
| | 23 | A0A0F7Z6K1_CROAD | Phosphatidate phosphatase | *Crotalus adamanteus* |
| | 22 | A0A0K8S0L2_CROHD | Iron-sulfur cluster assembly 1, mitochondrial-like protein | *Crotalus horridus* |
| | 20 | A0A0F7ZC99_CROAD | Charged multivesicular body protein | *Crotalus adamanteus* |

| | | | | |
|---|---|---|---|---|
| | 18 | A0A1W7RDB4_AGKCO | Sialic acid synthase-like protein | *Agkistrodon contortrix contortrix* |
| | 17 | A0A1W7RF54_AGKCO | NudC domain-containing protein 2 | *Agkistrodon contortrix contortrix* |
| | 16 | A0A1W7RHL5_AGKCO | Endoplasmic reticulum-Golgi intermediate compartment protein-2 like protein | *Agkistrodon contortrix contortrix* |
| | 15 | T1E4Y0_CROHD | Secernin-3-like protein | *Crotalus horridus* |
| | 15 | A0A0B8RW51_CROHD | Cytochrome P450 2j5-like protein | *Crotalus horridus* |
| | 15 | A0A0K8RXU5_CROHD | Tubulin-glutamate carboxypeptidase | *Crotalus horridus* |
| | 13 | A0A194AQ76_SISMB | C-type lectin 15 | *Sistrurus miliarius barbouri* |
| FG6 | 54 | J3RZT5_CROAD | Keratin, type I cytoskeletal 19 | *Crotalus adamanteus* |
| | 52 | A0A194APM7_9SAUR | C-type lectin 9d | *Agkistrodon piscivorus* |
| | 49 | A0A0B8RV71_CROHD | Keratin, type II cytoskeletal cochleal-like | *Crotalus horridus* |
| | 35 | A0A1W7REY6_AGKCO | Sister chromatid cohesion protein PDS5 B | *Agkistrodon contortrix contortrix* |
| | 32 | A0A0F72BJ1_CROAD | FAS-associated factor 1 | *Crotalus adamanteus* |
| | 30 | A0A0F7ZAB1_CROAD | Keratin, type I cytoskeletal 18-like protein | *Crotalus adamanteus* |
| | 29 | VM2_BITAR | Disintegrin bitistatin | *Bitis arietans* |

FG1 to FG6 were cut from the gel and subjected to in-gel digestion with trypsin. Analysis was performed using an Easy-nLC Proxeon nanoHPLC system coupled to an LTQ-Orbitrap Velos. The results obtained were compared with the "*Bitis*" and "Snakes" database downloaded from Uniprot (Taxid: 8570).
